# A fast and low-cost genotyping method for hepatitis B virus based on pattern recognition in point-of-care settings

**DOI:** 10.1038/srep28274

**Published:** 2016-06-16

**Authors:** Xianbo Qiu, Liuwei Song, Shuo Yang, Meng Guo, Quan Yuan, Shengxiang Ge, Xiaoping Min, Ningshao Xia

**Affiliations:** 1 College of Information Science and Technology, Beijing University of Chemical Technology, Beijing 100029, China; 2 National Institute of Diagnostics and Vaccine Development in Infectious Diseases, Xiamen University, Xiamen 361005, China; 3 Xiamen Innodx Biotech Company, Ltd., Xiamen 361026, China

## Abstract

A fast and low-cost method for HBV genotyping especially for genotypes A, B, C and D was developed and tested. A classifier was used to detect and analyze a one-step immunoassay lateral flow strip functionalized with genotype-specific monoclonal antibodies (mAbs) on multiple capture lines in the form of pattern recognition for point-of-care (POC) diagnostics. The fluorescent signals from the capture lines and the background of the strip were collected via multiple optical channels in parallel. A digital HBV genotyping model, whose inputs are the fluorescent signals and outputs are a group of genotype-specific digital binary codes (0/1), was developed based on the HBV genotyping strategy. Meanwhile, a companion decoding table was established to cover all possible pairing cases between the states of a group of genotype-specific digital binary codes and the HBV genotyping results. A logical analyzing module was constructed to process the detected signals in parallel without program control, and its outputs were used to drive a set of LED indicators, which determine the HBV genotype. Comparing to the nucleic acid analysis to HBV viruses, much faster HBV genotyping with significantly lower cost can be obtained with the developed method.

Regarded as one of the most serious public health problems in the world, the hepatitis B virus (HBV) related diseases including hepatitis, liver cirrhosis (LC) and hepatocellular carcinoma (HCC) extraordinarily threaten worldwide patients’ lives and cause more than one million deaths annually^1^,^2^. There are 10 recognized different HBV genotypes (A–J) prevailing globally, and among them, genotypes A/D are prevalent in North America/Europe and B/C in Asia^3^. In China, genotype A, B, C and D are identified with dominant genotypes B (36%) and C (51%)^4^. Long-term clinical studies show that the HBV genotype contributes to the risk of liver disease progression (LC and HCC), the rate of chronicity and especially the antiviral treatment response^5^,^6^. For example, from the historical virological responses and HBV surface antigen (HBsAg) losses, it is found that HBV genotype influences the on-treatment HBsAg kinetics and end-of-treatment (EOT) HBsAg levels. Therefore, the response-guided treatment (RGT) of chronic hepatitis B (CHB) could be ameliorated and improved by genotype-specific monitoring timeframes and EOT thresholds^7^. Therefore, similar to breast density classification for evaluation of the risk of breast cancer^8^, rapid determination of the HBV genotype is able to provide important information for the disease progression and the long-term CHB clinical antiviral treatment.

Many methods, such as direct genome sequencing^9^, reverse hybridization or line probe assay (LiPA)^10^, restriction fragment polymorphism (RELP)^11^, multiplex nested polymerase chain reaction (PCR)^12^, oligonucleotide microarray chips^13^, and real-time PCR^14^, for determining the HBV genotype have been developed. Most the existing methods including the recently reported genotyping method based on microfluidic loop-mediated isothermal amplification (LAMP) chip and giant magnetoresistive (GMR) sensors^15^ determine the HBV genotype through nucleic acid analysis. Due to the essential sample preparation step for DNA extraction, the above methods in whole are time-consuming, high cost and inconvenient especially because the involvement of DNA amplification.

Immunochromatographic assay, also known as lateral flow immunoassay, with its characteristics of low-cost, easy-to-use, simplicity, rapidness and high sensitivity, has been widely used in rapid or screening test in the point of care settings^16^,^17^. Once the sample is applied onto the strip, it will be wicked along the lateral flow strip by capillary force, and a few minutes later, the result becomes readable by eyes, or an optical reader^18^,^19^. Using the sandwich^20^ or competitive assay^21^, lateral flow strips are able to detect the presence of antigens or antibodies both in qualitative and quantitative forms.

In order to simplify the trivial procedure of HBV genotyping, for POC diagnostics, we developed a fast HBV genotyping method with a concise digital classifier based on a digital genotyping model and a one-step fluorescent lateral flow immunoassay (LFIA) strip functionalized with genotype-specific monoclonal antibodies (mAbs) on multiple capture lines. With the digital classifier, fluorescence signals from multiple capture lines were detected and processed in parallel by a logical analyzing module in the form of pattern recognition without any microcontrollers. Without program control, HBV genotyping result was represented by one group of genotype-specific digital binary codes. Straightforward HBV genotyping result can be obtained from the states of a group of LED indicators with a companion decoding table. The utility and performance of the POC diagnostics method for HBV genotyping were fully evaluated.

## Results

Before tests, all the serum samples collected from a cohort of more than hundred CHB patients were successfully subject to HBV genome sequence analysis for validation. A global HBV genotyping panel (BBI, WWHD301) was also used for verification test. The panel included 20 undiluted positive plasma samples from different individuals in 10 countries with totally eight identified HBV genotypes. The HBsAg levels were quantified with the Architect HBsAg assay (Abbott Laboratories, Abbott Park, IL, USA; detection range, 0.05–250 IU/mL).

For each immunochromatographic test with lateral flow strip, 80 μL sample (serum or plasma) is applied onto the sample pad, the dried conjugates and viral surface antigens are re-mobilized, and the resulted immune complexes in the samples are then captured if possible by the four antibodies based on the viral antigen genotypes when they migrate past the four capture lines respectively on the nitrocellulose membrane. Meanwhile, different reactions occur on both the S line and the control (CT) line respectively for positive control and assay justification. After 20 min when the immunochromatographic assay is completed, the strip in a cassette is putted into the classifier for test. Once the classifier is powered on, it will read the fluorescent signals from the multiple capture lines on the strip in parallel, and then the signals will be processed and analyzed until the genotyping result is provided through the states (On/Off) of a group of LED indicators. And finally, the genotyping result can be easily identified by just looking into the companion decoding table which defines all the possible pairing cases between the states of a group of LED indicators and the HBV genotyping results.

First, total 12 HBV positive serum samples chosen from the sample bank of CHB patients, of which each 3 respectively for genotype A, B, C, and D with high (40–80 IU/mL), moderate (20–40 IU/mL) and low (10–20 IU/mL) HBsAg level concentrations, were tested by the developed classifier with LFIA. As shown in Table 1, experimental results showed that all the serum samples can be successfully differentiated by the digital classifier. All the tests were repeated more than triple times, and similar experimental results were achieved. To determine the sensitivity of the developed classifier with LFIA, a series of experiments with further dilution of the positive serum samples from 100 IU/mL to 0 IU/mL were conducted. It was found that for effective differentiation of HBV genotypes A, B, C and D with high sensitivity and specificity, as show in Table 2, the analytical sensitivities of the digital classifier with LFIA for detecting HBsAg were respectively around 3.13, 6.25, 6.25 and 12.5 IU/mL, which met the requirement for clinical application. With the available test samples, it is found that the developed method works well when the upper concentration reaches around 100,000 IU/mL.

The digital classifier with LFIA was further evaluated with the detection of a global HBV genotyping panel (BBI, WWHD301). As show in Table 3, among all samples, two samples (WWHD301-01 and WWHD301-19) with very low HBsAg levels (<1.5 IU/mL) were regarded as an “HBsAg-negative” diagnosed by the digital classifier. HBV genome sequence analysis of the two HBV negative serum samples confirmed that they were free of HBV, which confirmed the results, “N”, by the developed classifier. For the other 18 samples, the obtained genotyping results by the digital classifier were consistent with the reference data. In details, the digital classifier detected 3 samples of genotype A, 1 sample of genotype B, 2 samples of genotype C, and 4 samples of genotype D. However, the digital classifier regarded 4 samples of genotype E as genotype D, and provided an “UN” result for 2 samples of genotype F, 1 sample of genotype G and 1 sample of genotype H. The reason for the fault diagnosis for the genotypes E, F, G and H is because the strip only has capture lines specifically for the genotypes A, B, C, and D, and does not have the antibodies for E, F, G and H. The above tests have been repeated more than triple times and consistent results were obtained. It can be concluded that the sensitivity of the developed method with the panel samples reaches 90.9% (10/11, 10 test samples are differentiated accurately from totally 11 test samples with genotypes A–D) compared with that of the sequence-based genotyping. Because no HBsAg negative samples are tested, the specificity of the developed method with the panel samples cannot be provided.

## Discussion

For the first time, we have shown a fast and low-cost HBV genotyping method with a digital classifier and one-step fluorescent LFIA strips in the point-of-care settings. For the existed HBV genotyping methods based on nucleic acid analysis, they normally involve a couple of different steps including sample preparation for DNA extraction, DNA amplification, DNA capturing and labeling, as a result, genotyping results are obtained with significantly long analysis time, high cost and inconvenience. For the newly developed HBV genotyping method with a concise digital classifier and easy-to-use one-step fluorescent LFIA strips, straightforward HBV genotyping results can be conveniently obtained within 20 minutes with extraordinarily low cost. Especially, for the purpose of POC diagnostics, the classifier instrument was assembled with components costing less than $100 due to the simplified signal detection and analysis methods as well as the concise and straightforward method for the showing of test results. With the newly developed POC diagnostics method, sensitive and accurate HBV genotyping results with reasonable repeatability can be obtained, which is beneficial to extend the application for HBV genotyping because of its low cost and high convenience.

In principle, genotyping for other diseases such as HIV could be performed with a similar method with a digital classifier and LFIA strips in the form of pattern recognition. A universal genotyping method for different diseases can be constructed with genotype-specific LFIA strips and a configurable digital classifier based on the technology of FPGA (Field Programmable Gate Array)^22^. The concept of HBV genotyping with a digital classifier and LFIA strips can be transplanted into other similar diagnostic systems, whose essential characteristics includes low cost, concise structure, small size and fast detection, for example, in the field of point-of-care test or smartphone-based portable biochemical detection.

## Methods

### Ethics statement

Written informed consent was obtained from each subject. Independent ethics committee approval was obtained from the Ethics Committee of the National Institute of Diagnostics and Vaccine Development in Infectious Diseases (NIDVD) of Xiamen University, China. All experiments were performed in accordance with relevant guidelines and regulations approved by the Ethics Committee of the NIDVD. All experimental protocols were approved by the Ethics Committee of the NIDVD. The methods were approved by the Ethics Committee of the NIDVD. The methods were carried out in accordance with the approved guidelines.

### Fabrication of lateral flow immunoassay strips

**Figure 1:**
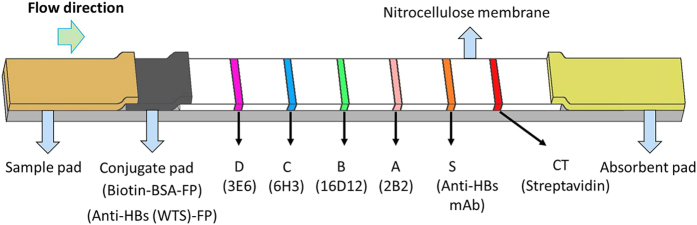
Schematics of lateral flow immunoassay strip for HBV genotyping.

Briefly, four HBV genotype-specific mAbs 2B2, 16D12, 6H3 and 3E6 were successfully developed and their sensitivity and specificity for detection of different HBV genotypes (A, B, C and D) were evaluated by enzyme-linked immunosorbent assay (ELISA). A fluorescent lateral flow immunoassay strip with multiple test lines and one control line was fabricated, as shown in Fig. 1.

The LFIA test strip was composed of a backing (Jieyi Biotechnology, Shanghai, China), sample pad (Shanghai Kinbio Tech Co., Ltd, Shanghai, China), conjugate pad (Shanghai Kinbio Tech Co., Ltd, Shanghai, China), nitrocellulose membrane (Millipore, Bedford, MA, USA) and absorbent pad (Jieyi Biotechnology, Shanghai, China). On the conjugate pad, an anti-HBsAg mAb (WTS (the name of the mAb); Wantai, Beijing, China) and biotinylated bovine serum albumin (BSA), both of which are conjugated to Fluoro-Max Fluorescent Nanoparticles (FP, Excite at 333 nm and emit at 613 nm, Thermo Scientific, Rockford, IL, USA), were stored in dry form after diluted in a blocking reagent with bovine serum albumin (BSA). On the nitrocellulose membrane, the genotype-specific mAbs 2B2, 16D12, 6H3 and 3E6, goat anti-HBsAg polyclonal (GAS) antibodies and streptavidin were respectively immobilized on the A, B, C, D, S and control (CT) capture lines as shown in Fig. 1. To ensure each capture line of the LF strip to be successfully detected by its own optical channel from the digital classifier, its positioning error in the detection window should be properly controlled. A custom jig was developed to facilitate the assembling of LF strips to guarantee positioning repeatability of capture lines.

### Protocol of lateral flow immunoassay

Briefly, once 80 μL sample (serum or plasma) is applied onto the sample pad, the dried conjugates and viral surface antigens, including L-HBsAg (containing the preS2 region), M-HBsAg (containing the preS2 region) and S-HBsAg (not containing the preS2 region) are re-mobilized, and the resulted L/M-HBsAg-WTS-FP immune complexes in the samples are then captured if possible by the 3E6, 6H3, 16D12 or 2B2 antibodies based on the viral antigen genotypes when they migrate past the capture lines (D, C, B and A, respectively) on the nitrocellulose membrane. Note that sandwich assay is adopted for each test line. Meanwhile, the S-HBsAg-WTS-FP immune complexes and biotinylated BSA-FP are respectively captured by the GAS antibody on the S line and streptavidin on the CT line. The assay is completed in 20 min before the fluorescent signals of capture lines can be detected with a fluorescence reader.

### HBV genotyping strategy

For the LFIA-based HBV genotyping, the fluorescent signal on the control line, i.e., CT-line with immobilized streptavidin, is used to guarantee that the sample has, indeed, travelled up the strips and the fluorescent reporters in mixed sample have not decayed. Therefore, for a valid test, the signal amplitude of the CT-line should be significantly higher than the background. Because the S-line is used to capture HBsAg for positive control, its fluorescent signal amplitude should also be significantly higher than the background for HBV positive tests. Unfortunately, parts of the four genotype-specific mAbs (3E6, 6H3, 16D12 and 2B2) for recognition of the genotype-specific variable regions of HBV preS2 exhibit cross-reactivity to heterogeneous HBV genotypes. As shown in Table 4, the 6H3 mAb reacts to both genotypes C and D, and the 3E6 mAb reacts to both genotypes D and A. Therefore, an intelligent HBV genotyping strategy, which defines the principle about how to determine the genotype from the detected signals, was developed based on the known bio-reactivity between mAbs and HBV genotypes as well as experimental results. In brief, when the fluorescence signal amplitude of the capture line A or B is significantly higher than the background and it is the maximum among four capture lines (A–D), genotype A or B can be identified. Otherwise, more intelligent analysis (to be explained in details in the subsection digital model of HBV genotype classifier) to fluorescence signals from the capture lines C and D is required because of the cross reactivity.

### System overview of HBV genotyping

**Figure 2:**
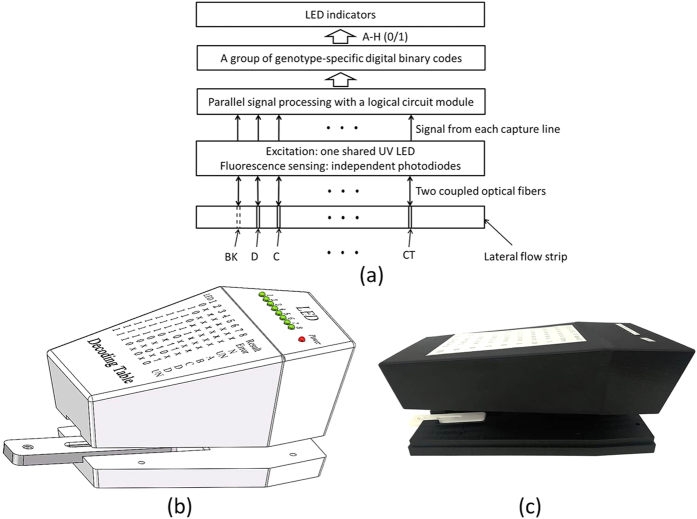
Digital classifier for HBV genotyping. (**a**) schematic depiction of its working principle, (**b**) 3D of the partly assembled digital classifier with an inserted strip cassette, and (**c**) picture of the actual device.

The digital classifier consisted of optical fibers, an UV LED, photodiodes, a logical analyzing module, LED indicators, and a decoding table. As shown in Fig. 2(a), the fluorescence signal of each capture line or the background was read by an independent photodiode with pre-amplifier (TSL257, Texas Advanced Optoelectronic Solutions Inc., TX, USA) through one optical fiber (1mm diameter, Hecho Ltd., China). A similar shining optical fiber was used to transmit UV light from a shared UV LED (365 nm, CUN66A1B, SEOUL VIOSYS, Korea). The logical analyzing module was made of a logical circuit consisting of comparators, amplifiers and multiplexers, and processed the detected signals from the capture lines in parallel according to the digital genotyping model (to be explained in the next subsection) without program control. The outputs of the logical circuit module were a group of genotype-specific digital binary codes (a–h) which were directly used to drive a set of LED indicators (1–8) respectively, e.g., 1 for LED indicator (1–8) on and 0 for LED off.

Without any microcontrollers, motors, liner moving stages, and display screens, a handheld, concise digital classifier (Fig. 2(b)) was assembled with components costing less than $100. The actual device was shown in Fig. 2(c). Once the LF strip cassette is inserted into the tray, the top and bottom parts of the digital classifier can be easily assembled to form a closed dark chamber for fluorescence detection. It takes just a few milliseconds for signal detection and analysis to be completed. A set of LED indicators (1–8) are used to exhibit the states of a group of genotype-specific digital binary codes from the logical circuit module. Therefore, from the states of a set of LED indicators (1–8) positioned on the panel of the digital classifier, the genotyping result can be easily identified.

### Digital model of HBV genotyping

**Figure 3:**
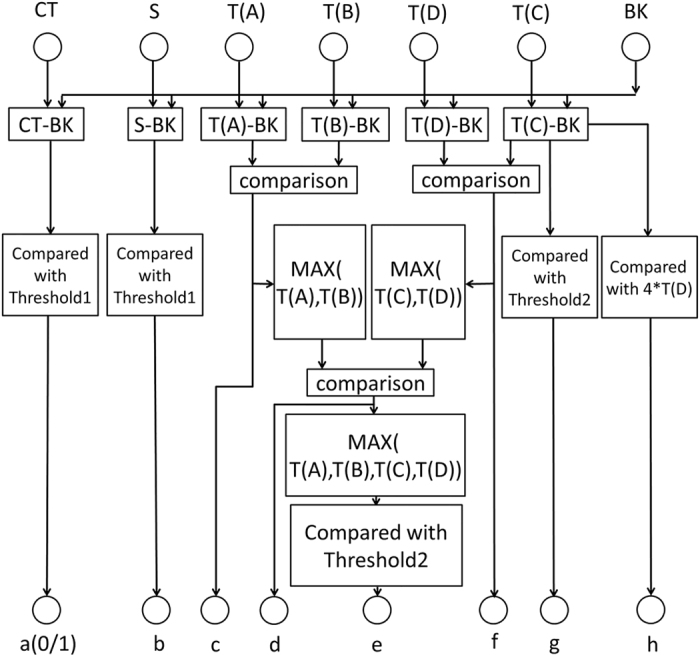
Digital model for HBV genotyping.

For HBV genotyping, the multiple fluorescent signal peaks of LF strips are analyzed by a digital model based on the HBV genotyping strategy in the form of pattern recognition. To perform HBV genotyping without any software algorithms and microcontrollers, the digital genotyping model should be developed in a way that all the detected signals from the capture lines and the background can be analyzed in parallel and each HBV genotyping result can be represented by a group of genotype-specific digital binary codes (0/1), as shown in Fig. 3.

The inputs of the genotyping model include detected fluorescence signals (CT, S, T(A), T(B), T(C), T(D) and BK) from both the capture lines (CT, S, A, B, C, and D) and the background (BK), and its outputs are a group of genotype-specific digital codes (a–h) in binary (0/1). After the original fluorescence signal from each capture line was subtracted by the background, all the signals are compared to others or the threshold value by following their own signal streams whose final outputs consisting of a group of genotype-specific digital binary codes. Two threshold values, which define the minimum of consistently recognizable fluorescent signal amplitudes subtracted by the background respectively for the CT/S lines and the capture lines A–D, were obtained from experiments. In the experiment, HBV positive samples with different HBsAg level concentrations including high (40–80 IU/mL), medium (20–40 IU/mL) and low (10–20 IU/mL) ones were tested to find two proper values respectively for the two thresholds. The experiments were repeated more than triple to obtain the consistent results.

Without any microcontrollers and memories, for the digital HBV genotyping model, the fluorescence signals must be processed in a parallel way because any temporary data cannot be stored. Accordingly, multiple signal streams were created inside the digital model. From the viewpoint of pattern recognition, the final states or outputs of multiple signal streams are related to a specific HBV genotype. In order to simplify the digital model, the final state or output of each signal stream was expressed by a binary code, e.g., 1 or 0, and all of them formed a group of genotype-specific digital binary codes.

Meanwhile, a companion decoding table (Table 5) was established to cover all possible pairing cases between the states of a group of genotype-specific digital binary codes and the HBV genotyping results. There totally have nine different cases (case 1–9) for possible states of a group of genotype-specific digital binary codes (a–h), which totally correspond to seven different genotyping results, i.e., “Error” for failure test, “N” for HBV negative test, “UN” for test with undifferentiated genotype, “A” for test with genotype A, “B” for test with genotype B, “C” for test with genotype C, and “D” for test with genotype D.

As shown in Table 1, “x” means that no matter the state of a digital code is 1 or 0, the genotyping result will be the same. With the above digital model, the pairing relationship between the states of a group of genotype-specific digital binary codes and the HBV genotyping results in Table 1 can be explained as following. For case 1, A = 0 means that fluorescence signal amplitude of the control line subtracted by the background is lower than the threshold 1, which corresponds to the failure test (Error). For case 2, A = 1 and B = 0 mean that LF test is successful while fluorescence signal amplitude of the S line subtracted by the background is lower than the threshold 1, which corresponds to the negative test (N). For case 3, A = 1, B = 1 and E = 0 mean that LF test with positive HBV sample is successful while the maximum fluorescence signal amplitude of the four capture lines (A–D) subtracted by the background is lower than the threshold 2, which corresponds to the undifferentiated genotype (UN). For case 4, A/B/C/D/E = 1 mean that LF test with positive HBV sample is successful while the fluorescence signal amplitude of the capture line A subtracted by the background is higher than the threshold 2 and it is the maximum among four capture lines (A–D), which corresponds to genotype A (A). For case 5, A/B/D/E = 1 and C = 0 mean that LF test with positive HBV sample is successful while the fluorescence signal amplitude of the capture line B subtracted by the background is higher than the threshold 2 and it is the maximum among four capture lines (A–D), which corresponds to genotype B (B). For case 6, A/B/E/F/H = 1 and D = 0 mean that LF test with positive HBV sample is successful while the fluorescence signal amplitude of the capture line C subtracted by the background is higher than the threshold 2 and it is the maximum among four capture lines (A–D), and the fluorescence signal amplitude of the capture line C is higher than four times (it was determined by experiments with different samples with high-low concentrations, and it was found that four times was able to provide more consistent results than others in the required detection range) of that of the capture line D, which corresponds to genotype C (C). For case 7, A/B/E/F = 1 and D/H = 0 mean that LF test with positive HBV sample is successful while the fluorescence signal amplitude of the capture line C subtracted by the background is higher than the threshold 2 and it is the maximum among four capture lines (A–D), and the fluorescence signal amplitude of the capture line C is lower than four times of that of the capture line D, which corresponds to genotype D (D). For case 8, A/B/E/G = 1 and D/F = 0 mean that LF test with positive HBV sample is successful while the fluorescence signal amplitude of the capture line D subtracted by the background is higher than the threshold 2 and it is the maximum among four capture lines (A–D), and the fluorescence signal amplitude of the capture line C is higher than the threshold 2, which corresponds to genotype D (D). Because the 3E6 mAb immobilized on capture line D did not react with either genotype B or genotype C but could effectively capture both genotype D (sensitivity: 1 IU/mL) and genotype A (sensitivity: 20 IU/mL), for case 8, different from case 7, it is unnecessary to compare signals between lines C and D. For case 9, A/B/E = 1 and D/F/G = 0 mean that LF test with positive HBV sample is successful while the fluorescence signal amplitude of the capture line D subtracted by the background is higher than the threshold 2 and it is the maximum among four capture lines (A–D), and the fluorescence signal amplitude of the capture line C is lower than the threshold 2, which corresponds to the undifferentiated genotype (UN).

In principle, to get rid of the decoding table, an alternative, much more complicated genotyping model than the developed one can be built by adding supplementary signal processing units to enable each genotyping result to be represented by just one specific binary code instead of a group of binary codes.

### Logical analyzing module of HBV genotyping

**Figure 4:**
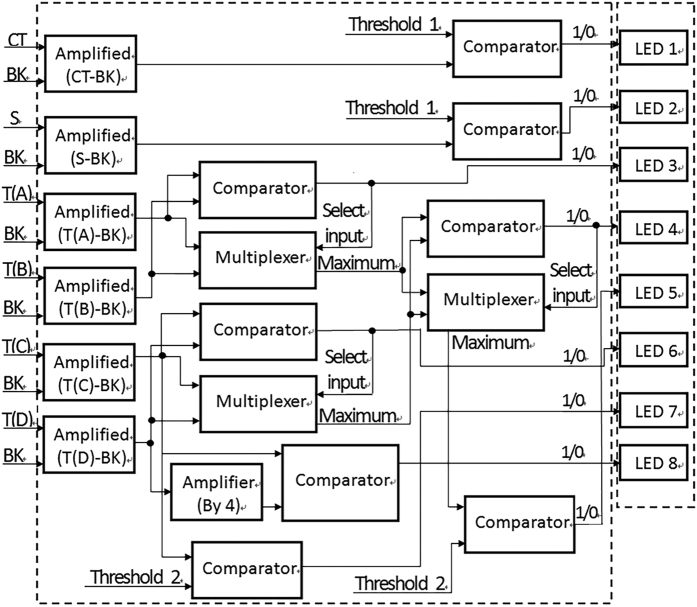
Schematics of the logical circuit module for parallel signal processing.

To perform HBV genotyping with a digital classifier with LFIA strips, the above digital model was realized by a logical analyzing module made from a logical circuit consisting of integrated chips to process the detected signals from capture lines in parallel. Similar to the digital model, the outputs of the logical circuit module were a group of genotype-specific digital binary codes (1/0) which were directly used to drive a set of LED indicators respectively, e.g., 1 for LED on and 0 for LED off. Figure 4 shows the schematics of the logical circuit module for parallel signal processing.

As shown in Fig. 4, similar to the digital model, after original fluorescence signal for each capture line (CT, S, T(A), T(B), T(C), T(D)) was subtracted by the background (BK) and then amplified, all the signals were further processed by an optimized logical analyzing circuit consisting of six instrumentation amplifier ICs (AD8421, Analog Devices, USA), an operational amplifier IC (LMV612, Texas Instruments, USA), 2 comparator ICs (LM3302, Texas Instruments, USA) and 2 multiplexer ICs (TS5A23159, Texas Instruments, USA). The select input of each multiplexer was controlled by the output of a comparator to switch on the larger one of two inputs. Two threshold values, which have been defined previously, were provided by a precision voltage reference (LM4120, National Semiconductor, USA) with two separate potentiometers. Finally, HBV genotyping result can be determined from the states of one set of LED indicators (1–8) with the above companion decoding table. With a logical circuit module for signal analysis in parallel, HBV genotyping could be performed without any microcontrollers, which reduced the system’s complexity and cost.

## Additional Information

**How to cite this article**: Qiu, X. *et al*. A fast and low-cost genotyping method for hepatitis B virus based on pattern recognition in point-of-care settings. *Sci. Rep.* **6**, 28274; doi: 10.1038/srep28274 (2016).

## Figures and Tables

**Table 1:** Test result with HBV positive serum samples.

Sample	a	b	c	d	e	f	g	h	Result
Genotype A(10–20 IU/mL)	1	1	1	1	1	x	x	x	A
Genotype B(10–20 IU/mL)	1	1	0	1	1	x	x	x	B
Genotype C(10–20 IU/mL)	1	1	x	0	1	1	x	1	C
Genotype D(10–20 IU/mL)	1	1	x	0	1	1	x	0	D
Genotype A(20–40 IU/mL)	1	1	1	1	1	x	x	x	A
Genotype B(20–40 IU/mL)	1	1	0	1	1	x	x	x	B
Genotype C(20–40 IU/mL)	1	1	x	0	1	1	x	1	C
Genotype D(20–40 IU/mL)	1	1	x	0	1	1	x	0	D
Genotype A(40–80 IU/mL)	1	1	1	1	1	x	x	x	A
Genotype B(40–80 IU/mL)	1	1	0	1	1	x	x	x	B
Genotype C(40–80 IU/mL)	1	1	x	0	1	1	x	1	C
Genotype D(40–80 IU/mL)	1	1	x	0	1	1	x	0	D

**Table 2:** Sensitivity with LFIA assisted by classifier for four genotypes (A–D).

Genotype	Sensitivity with LFIA assisted by digital classifier
A	3.13 IU/mL
B	6.25 IU/mL
C	6.25 IU/mL
D	12.5 IU/mL

**Table 3:** Test result with samples from a global HBV genotyping panel.

Sample	Reference Genotype	LFIA assisted by digital classifier
WWHD301-01	A	N
WWHD301-02	A	A
WWHD301-03	A	A
WWHD301-04	E	D
WWHD301-05	A	A
WWHD301-06	B	B
WWHD301-07	C	C
WWHD301-08	C	C
WWHD301-09	D	D
WWHD301-10	D	D
WWHD301-11	D	D
WWHD301-12	D	D
WWHD301-13	E	D
WWHD301-14	E	D
WWHD301-15	E	D
WWHD301-16	F	UN
WWHD301-17	F	UN
WWHD301-18	G	UN
WWHD301-19	G	N
WWHD301-20	H	UN

**Table 4:** Cross-reactivity of mAbs with four genotypes (A–D).

mAb	A	B	C	D
6H3	N	N	Y	Y
3E6	Y	N	N	Y

**Table 5:** Decoding table for HBV genotyping.

Digital Code	a	b	c	d	e	f	g	h	Result
Case1	0	×	×	×	×	×	×	×	Error
Case2	1	0	×	×	×	×	×	×	N
Case3	1	1	×	×	0	×	×	×	UN
Case4	1	1	1	1	1	×	×	×	A
Case5	1	1	0	1	1	×	×	×	B
Case6	1	1	×	0	1	1	×	1	C
Case7	1	1	×	0	1	1	×	0	D
Case8	1	1	×	0	1	0	1	×	D
Case9	1	1	×	0	1	0	0	×	UN
